# Evaluation of the Partial Replacement of Dietary Fish Meal With Fermented or Untreated Soybean Meal in Juvenile Silver Barb, *Barbonymus gonionotus*

**DOI:** 10.3389/fnut.2021.733402

**Published:** 2021-11-01

**Authors:** Halima Jahan, Israt Jahan Tumpa, Wafaa A. Qasem, Mohammad Moniruzzaman, Mst. Arzu Pervin, Rabeya Akter, Abdelwahab Omri, Taesun Min, Zakir Hossain

**Affiliations:** ^1^Department of Fisheries Biology and Genetics, Faculty of Fisheries, Bangladesh Agricultural University, Mymensingh, Bangladesh; ^2^Department of Fisheries Biology and Genetics, Sher-e-Bangla Agricultural University, Dhaka, Bangladesh; ^3^Department of Surgery, Mubarak Al Kabeer Hospital, Hawally, Kuwait; ^4^Community Medicine Department, Faculty of Medicine, Kuwait University, Kuwait City, Kuwait; ^5^Department of Animal Biotechnology, Jeju International Animal Research Center, Sustainable Agriculture Research Institute (SARI), Jeju National University, Jeju-si, South Korea; ^6^The Novel Drug and Vaccine Delivery Systems Facility, Department of Chemistry and Biochemistry, Laurentian University, Greater Sudbury, ON, Canada

**Keywords:** fish meal, soybean meal, digestive enzymes, antioxidant enzymes, blood chemistry

## Abstract

Fish meal (FM) has excellent protein and lipid profile. However, FM is losing its acceptability and substituted with plant protein due to FM has high price, high demand, and sustainability issues in global aquaculture production. In this study, experimental diets were prepared by substituting FM with fermented soybean meal (FSM) or normal and untreated soybean meal (SM) to assess the effects on growth, hematology, innate immunity, gut physiology, and digestive enzyme activities in juvenile silver barb, *Barbonymus gonionotus*. Five diets, that is, 40% FM (FM 40), 20% FM + 20% FSM (FM 20 + FSM 20), 20% FM + 20% SM (FM 20 + SM 20), 40% FSM (FSM 40), and 40% SM (SM 40) were fed to the fish two times daily for 90 days. After 90 days of feeding trial, FM 40, FM 20 + FSM 20, and FM 20 + SM 20 diet groups showed significantly higher weight gain (WG) and specific growth rate (SGR) compared to the FSM 40 and SM 40 diets. Hepatosomatic index (HSI) and viscerosomatic index (VSI) were significantly higher in fish fed with the FSM 40 and SM 40 diets than those of fish fed with the FM 40 diet. Hematocrit, hemoglobin, and erythrocyte count were significantly lower in fish fed with the SM 40 diet compared to fish fed with the FM 40 and FM 20 + FSM 20 diets. Superoxide dismutase and catalase activities in the liver were significantly higher in fish fed with the SM 40 diet compared to fish fed with the FM 40 diet. However, serum thiobarbituric acid reactive substances in fish fed with the experimental diets were unaltered. Fish showed significant reduction of villus height (Vh) in the anterior and posterior intestine of fish fed with the FSM 40 and SM 40 diets, whereas muscular thickness was opposite to the findings of Vh. Digestive enzyme activities in intestine were significantly higher in fish fed with the FM 40 diet compared to those in the SM 40 diet. The results of the present study revealed that the 50% of FM can be replaced by FSM or SM as a source of protein without affecting the growth of juvenile silver barb.

## Introduction

Fish meal (FM) is recognized for its high-quality protein, digestible amino acids, and palatability among other feed ingredients used in the formulated diet of fish, but its high price and imbalance in demand and supply emphasizes the significance of utilizing plant proteins as an alternative of FM for the sustainable aquaculture (1, 2). Plant proteins are focusing for use in aquaculture production, as the promising feed ingredients in the diet development for fish by replacing FM. In particular, the FM replacement with economically viable and ecologically friendly soybean meal (SM) is a good alternative (3). SM is used in fish feeds due to its high energy content, high protein digestibility, excellent amino acid, and fatty acid profile, providing vitamins, low cost, feed attractant, availability, and steady supply (4, 5). Fermented SM (FSM) enhances the biodegradation of anti-nutritional factors (ANFs), increases its protein content, and facilitates antioxidant activity, nutrients digestibility, and immune function in animals through the production of probiotics and prebiotics (6, 7). Considerable success in partial replacement of FM by dietary SM without affecting growth performance has been reported in Atlantic salmon (8), Black sea bream (9), Senegalese sole (10), and Atlantic cod (11). The complete replacement of FM by SM can cause size reduction of the mucosal folds, swelling of the lamina propria and subepithelial mucosa, loss of supranuclear vacuoles in the intestinal epithelium, and decreased absorptive vacuoles in the enterocytes (12). However, partial dietary supplementation of SM has been reported to have positive effects on digestive physiology, antioxidant enzyme activities (13), and hematological conditions (14) in fish.

As silver barb, *Barbonymus gonionotus*, is an omnivore species and is evident to have a considerable amount of plant protein in the diet with a wide range of stress tolerances, and it is used as a model animal in this study. Since soy protein is a very nutritive ingredient and is known for its protective effects on health and gastrointestinal disorders (15), it is safe to combine soy protein with other animal proteins at a certain level (16). Hence, in this study, 50% of FM was replaced with FSM or SM and has been selected as a source of protein of the experimental diets along with other ingredients and the binding agent to hold the feed for this model animal. The aim of the present study was to investigate the impacts of replacing FM with FSM or SM on growth, hematology, antioxidant activities, gut morphology, and digestive enzyme activities of *B. gonionotus*.

## Materials and Methods

### Fermentation of Soybean Meal

A mixture of ground and brown sugar was dipped in water at the ratio of 3:1:10 (soybean: brown sugar: water) for 12 h. Sugar (10%) and *Lactobacillus paracasei* HII02 starter (10%) were added to the untreated SM and then the mixture was incubated for 20 days at 30°C. Finally, the fermented samples were dried at room temperature and crushed to use as a fermented soybean meal (FSM) feed ingredient.

### Feed Formulation and Proximate Composition of Diets

Five experimental diets, that is, FM 40 (40% inclusion of FM in the diet), FM 20 + FSM 20 (40% of FM substituted with 20% FM and 20% FSM, hereafter, 50% replacement of FM with FSM), FM 20 + SM 20 (40% of FM substituted with 20% FM and 20% SM, hereafter, 50% replacement of FM with SM), FSM 40 (40% of FM substituted with 40% of FSM, hereafter, 100% replacement of FM with FSM), and SM 40 (40% of FM substituted with 40% of SM, hereafter, 100% replacement of FM with SM) were formulated using rice bran, wheat flour, FM, maize meal, FSM, SM, vitamin premix, mineral premix, and cod liver oil as ingredients (Table 1). Feeds were prepared according to the standard methods; briefly, ingredients were ground finely by a grinding machine and sieved with a fine mesh net. After sieving, the required amount of each ingredient was weighed as per formulae using the AND-GULL precision electronic balance (Mode-E600 Dual, made in UAE). The proximate composition of feed ingredients and formulated feeds are given in Table 2. After mixing all the ingredients, the required amount of water (at least 10%) was added to the feeds to make dough using a mixer. Then the dough feeds were passed through a pelleting machine to obtain pellet feeds (2 mm diameter) and dried in sunlight. The diets were broken up and sieved into the appropriate pellet size, allowed to store in the plastic bag in air tight condition, and kept in the refrigerator for later use. Moisture, crude protein, crude lipid, and ash contents of the dietary ingredients and feeds were analyzed by using the method of AOAC (17). Briefly, moisture contents (1 g for each sample) of ingredients and diets were determined by drying at 135°C for 2 h. Crude protein content (0.1 g) was determined using the Kjeldahl method (*N* × 6.25) after acid digestion, distillation, and titration of the samples. For crude lipid, 1 g of each sample was used in Soxhlet extraction (Soxtec system, Tecator AB, Hoganas, Sweden). Ash content (1 g for each sample) was estimated by a muffle furnace at 550°C for 4 h. Crude fiber contents were determined by a fiber analyzer (ANKOM 200 Fiber Analyzer, New York, NY, USA). Carbohydrate contents were measured using the following formulae:


$$\begin{array}{l} Carbohydrates(\%)=100-(moisture+lipids+proteins\\ \;+ash)\%. \end{array}$$

**Table 1:** Feed formulation of the experimental diets with different ingredients (g kg^−1^ of dry matter basis).

**Ingredients**					
	**FM 40**	**FM 20 + FSM 20**	**FM 20 + SM 20**	**FSM 40**	**SM 40**
Wheat flour	100	100	100	100	100
Wheat bran	150	150	150	150	150
Rice bran	200	200	200	200	200
Maize meal	130	130	130	130	130
Fish meal	400	200	200	0	0
Fermented soybean meal	0	200	0	400	0
Soybean meal	0	0	200	0	400
Vitamin premix	5	5	5	5	5
Mineral premix	5	5	5	5	5
Cod liver oil	10	10	10	10	10

*FM 40, FM 20 + FSM 20, FM 20 + SM 20, FSM 40, and SM 40 indicate diets containing 40% fish meal (FM), 20% FM + 20% fermented soybean meal (FSM), 20% FM + 20% soybean meal (SM), 40% fermented soybean meal, and 40% soybean meal, respectively*.

**Table 2:** Proximate composition of feed ingredients and formulated diets (% dry matter basis)^a^.

	**Moisture**	**Crude lipid**	**Crude protein**	**Ash**	**Crude fiber**	**Carbohydrate**
**Ingredients**						
Wheat flour	15.4 ± 1.3	2.2 ± 0.2	9.0 ± 0.8	1.7 ± 0.1	0.8 ± 0.1	72.6 ± 3.3
Rice bran	18.8 ± 2.5	10.6 ± 1.1	12.0 ± 1.5	7.4 ± 0.8	7.2 ± 0.7	44.1 ± 3.3
Wheat bran	16.0 ± 1.6	4.8 ± 0.5	14.5 ± 1.8	2.9 ± 0.3	6.9 ± 0.6	54.0 ± 4.3
Maize meal	15.3 ± 1.8	3.2 ± 0.2	16.1 ± 1.9	7.6 ± 0.6	6.7 ± 0.5	51.1 ± 3.1
FSM	12.8 ± 1.1	2.3 ± 0.3	48.5 ± 2.7	4.5 ± 0.4	5.9 ± 0.5	24.0 ± 2.6
SM	14.4 ± 1.2	4.4 ± 0.3	44.5 ± 2.7	5.3 ± 0.4	6.5 ± 0.5	26.0 ± 2.6
Fish meal	10.2 ± 1.2	8.5 ± 1.0	59.6 ± 3.2	18.0 ± 2.2	1.8 ± 0.1	0.9 ± 0.1
**Diets**						
FM 40	40.4 ± 3.3	3.2 ± 0.2	30.1 ± 2.0	3.8 ± 0.1	4.2 ± 0.2	18.4 ± 1.8
FM 20 + FSM 20	42.6 ± 2.8	3.4 ± 0.3	28.1 ± 2.3	4.2 ± 0.2	4.5 ± 0.3	17.0 ± 1.6
FM 20 + SM 20	40.5 ± 3.1	3.5 ± 0.1	26.0 ± 2.5	3.9 ± 0.2	4.7 ± 0.4	17.6 ± 1.9
FSM 40	41.5 ± 3.5	3.9 ± 0.3	25.4 ± 2.1	4.1 ± 0.2	4.9 ± 0.2	17.9 ± 2.0
SM 40	42.3 ± 3.5	3.9 ± 0.4	24.6 ± 2.6	4.3 ± 0.4	5.9 ± 0.5	18.5 ± 2.1

a *Values are presented in the mean ± SD of triplicate groups (n = 3) of samples*.

*FSM, fermented soybean meal; SM, soybean meal*.

### Experimental Design and Condition

To study the effects of SM or FSM in replacement of FM in juvenile silver barb, *B. gonionotus*, the feeding trial was conducted in a semi-recirculating system consisting of 15 cisterns (2.44 × 1.23 × 0.46 m) inside the Mini Hatchery Complex of Department of Fisheries Biology and Genetics, Bangladesh Agricultural University, Mymensingh, Bangladesh. Triplicate cisterns (15 cisterns) have corresponded to each experimental treatment group. Juvenile silver barb (*B. gonionotus*) were collected from Bangladesh Fisheries Research Institute, Mymensingh, Bangladesh. Each cistern was stocked with 60 juveniles of silver barb having initial weight and length of 8.8 ± 1.2 g (mean ± SD) and 8.6 ± 0.9 cm, respectively, and reared for 90 days. Fish were fed twice a day at 9:00 A.M. and 5:00 P.M. Formulated feeds were supplied near the shelter made for the fish, and the unused feedstuff, debris, and feces were removed by siphoning on a daily basis. The whole cisterns were emptied, cleaned, and washed with potassium permanganate every 30 days interval to minimize the fouling from the provided feeds and metabolic wastes. While 20% of each of the cistern's water was exchanged every day through the inlet and outlet systems. Fortnightly (every 15 days after) sampling was done to assess the growth and health condition of the fish. During sampling, 10 fish from each cistern were caught randomly with the help of a scoop net by lowering the water level and immediately anesthetized with 100 mg L^−1^ MS-222 (tricaine methanesulfonate). Then the weight and length of each fish were taken by six-digit sensitive electric balance (Mode-E600 Dual, UAE) and measuring scale, respectively. The average temperature, dissolved oxygen, and pH of the experimental cisterns were maintained at 26.0 ± 1.0°C, 9.0 ± 0.1 ppm, and 7.5 ± 0.5, respectively. All experimental trials used in this study followed the ethical guidelines approved by the Animal Welfare and Experimental Ethics Committee (AWEEC/BAU/2020-21) of Bangladesh Agricultural University, Mymensingh, Bangladesh.

### Growth Parameters

The weight of the individual body of fish, liver, and digestive tract were measured to calculate the growth performance and somatic indices of fish fed with the experimental diets according to the following formula:


$$\begin{array}{l} \;Weight\;gain(\%)=(Final\;weight\\ \;-Initial\;weight)/Initial\;weight\\ \;\times \;100\\ Specific\;growth\;rate\;(SGR\%,day^{\text{-1}})=\{(ln(Final\;weight)\\ \;-ln(Initial\;weight))\\ \;/Duration\;(90\;days)\}\times \;100\\ \;Hepatosomatic\;index\;(HSI\%)=(Liver\;weight/Body\;weight)\\ \;\times \;100.\\ \;Viscerosomatic\;index\;(VSI\%)=(Visceral\;weight/Body\;weight)\\ \;\times \;100. \end{array}$$

### Hematological Parameters

After acclimatization, fish were kept in three groups in triplicates to comprehend the changes in the morphology of the blood cells. Blood smears were processed on glass slides from fresh non-heparinized blood and then followed by air-drying, methanol fixation, and staining with the Wright's Giemsa. Blood corpuscles were then examined by immersion oil microscopy. Photographs were taken with the help of Intel Pentium Q3X computer-attached microscope under 400 × lens (Olympus-CX41, Tokyo, Japan). Red blood cells (RBCs) were counted according to the modified method of Math et al. (18). Briefly, blood collected from the caudal vein was taken into the RBC pipette (0.5 ppm), and the excess blood was removed with the cotton. Hayem's solution was taken up to 101 marks with the pipette. The solution of the pipette was then assorted thoroughly by an “8” knot motion. Consequently, two to three drops of fluid were expelled, and the next drop was placed on the chamber and the cover slipped. The cell was counted using a microscope.

The number of RBC per cubic mm was calculated by using the following formula:


$$\begin{array}{l} Total\;RBC(\times 10^{6}ml^{\text{-1}})\;=\;(number\;of\;cells\;\times \;dilution\;factor\\ \;\times \;depth\;factor)/(total\;number\;of\\ \;small\;squares\;\times \;16) \end{array}$$

Hemoglobin (Hb) (g dl^−1^) was determined with Hb strips using a kit (EasyMate® Blood Testing Meter, CEI Technology, Beijing, China). Hematocrit (Hct) was measured following the method of Nelson and Morris (19), utilizing the microcentrifuge and microhematocrit reader. The value was expressed based on the percentage of the RBC concentration.


$$\begin{array}{l} Hct\left(\%\right)=\left(L_{1}/L_{2}\right)\times \;100 \end{array}$$

where L_1_ is the height of the RBC column, and L_2_ is the total length of the column (RBC + plasma + buffer coat) in mm and expressed in %.

The blood samples were collected from six fish of each group by puncturing the vein by using a syringe in the caudal region. Blood glucose was measured by a blood glucose meter (HEALTH ASSURE®, Taipei, Taiwan) using an aliquot of the collected fresh blood.

### Antioxidant Enzyme Activities

Superoxide dismutase (SOD), glutathione peroxidase (GPx), and catalase (CAT) activities of the liver were determined following the methods of commercial kits (Cell Biolabs Inc., San Diego, CA, USA) as described by Pervin et al. (13) and Qasem et al. (20). Briefly, an appropriate amount of liver tissue sample, chromogen solution, 10 × SOD assay buffer, and Deionised water (DI) water were pipetted to the 96-well plate; 10 μl of pre-diluted 1 × xanthine oxidase solution was added to each well and incubated for 1 h at 37°C. The optical density was measured on a microplate reader at 490 nm to determine the SOD activity. For the CAT activity, 25 μl of the 10-Acetyl-3,7-Dihydroxyphenoxazine (ADHP)/Horseradish Peroxidase (HRP) working solution was added to the mixture of each well and incubated on a shaker for 30 min at 37°C. The optical density was determined with a fluorescence microplate reader with excitation at 530–570 nm range and emission at 590–600 nm. For the GPx activity, 100 μl of the prepared GPx, 25 μl of the 1 × Nicotinamide Adenine Dinucleotide Phosphate Hydrogen (NADPH), 50 μl of the 1 × chromogen, and 25 μl of the glutathione disulfide solution were added to the 96-well plates and determined the optical density at 405 nm at 1-min intervals for 10 min.

### Thiobarbituric Acid Reactive Substances

Six fish from the cistern of each dietary group were caught and immediately anesthetized using MS-222 at the end of the feeding trial. The blood was collected from the caudal vein and centrifuged at 7,500 × *g* at 4°C for 5 min to obtain the supernatant for measuring the thiobarbituric acid reactive substance (TBARS). The TBARS was determined using the method of Mohebbi et al. (21) modified by Pervin et al. (13). Briefly, the optical density of the supernatant was determined against the reference blank at 535 nm in a spectrophotometer. The rate was expressed in terms of μmol of TBARS formed/hr/mg protein using a molar extinction coefficient of 1.56 × 10^5^ m^−1^ × cm^−1^.

### Histomorphology of Digestive Tract

Histomorphological analysis was done following the standard procedures; briefly, the anterior and posterior intestines of each fish were preserved in 10% buffered formalin. Cleaning, infiltration, and dehydration processes were carried out using a series of alcohol in increasing concentrations. Paraffin-embedded blocks were cut by microtome knife at 5 μm size and left the sections into a water bath at a temperature of 40°C. The sections were stained routinely with hematoxylin and eosin as per the schedule. The stained sections were mounted on the glass slide with Canada balsam (mountant) and photographed under a compound microscope (Olympus, CX41RF, Tokyo, Japan). The villus height (Vh) and muscular thickness were measured following the criteria suggested by Wang et al. (22).

### Digestive Enzyme Activities in Fish

The enzymes, such as protease, amylase, and lipase activities, were measured following the casein hydrolysis method of Walter (23). Enzyme hydrolysis of starch based on the method of Swain et al. (24) and Albro et al. (25), respectively, with slight modification by Pervin et al. (13). Briefly, the optical density of the supernatant was determined at 280, 690, and 406 nm for protease, amylase, and lipase, respectively, in a spectrophotometer. The specific activity was expressed as unit/mg protein.

### Statistical Analysis

All the data were analyzed by one-way ANOVA using SPSS version 17.0 software (SPSS Inc., Chicago, IL, USA) to test for the effects of the dietary treatments. When a significant treatment effect was observed, Tukey's honestly significant difference (HSD) *post-hoc* test was used to compare the differences among the treatment means (*p* < 0.05).

## Results

### Fish Growth and Somatic Indices

The average WG and somatic indices of juvenile silver barb fed with the test diets are presented in Table 3. WG and SGR were significantly increased (*p* < 0.05) in FM 40, FM 20 + FSM 20, and FM 20 + SM 20 diet groups compared with FSM 40 and SM 40 diet groups. HSI was significantly higher (*p* < 0.05) in fish fed with the FM 40 and FM 20 + FSM diets compared with the FSM 40 and SM 40 diet groups, whereas on the contrary VSI augmented against FM reduction and showed significantly higher in SM 40 and FSM 40 diets.

**Table 3:** Growth performances and somatic indices of *Barbonymus gonionotus* fed with different experimental diets for 90 days^a^.

**Parameters**	**Diets**				
	**FM 40**	**FM 20 + FSM 20**	**FM 20 + SM 20**	**FSM 40**	**SM 40**
WG (%)	87.3 ± 9.5^a^	80.4 ± 6.7^a^	78.7 ± 6.5^a^	67.4 ± 5.8^b^	52.0 ± 5.1^b^
SGR (% d^−1^)	6.9 ± 0.3^a^	6.4 ± 0.2^a^	5.7 ± 0.3^b^	5.1 ± 0.1^c^	4.9 ± 0.2^c^
HSI (%)	2.4 ± 0.4^a^	2.4 ± 0.2^a^	1.9 ± 0.3^ab^	1.3 ± 0.3^b^	1.3 ± 0.4^b^
VSI (%)	2.7 ± 0.2^a^	3.1 ± 0.2^ab^	3.4 ± 0.4^ab^	3.7 ± 0.3^b^	3.8 ± 0.4^b^

a *Values are presented with the mean ± SD of triplicate groups (n = 3) of fish where the values in each row with different superscripts are significantly different (p < 0.05)*.

*SGR, specific growth rate; HSI, hepatosomatic index; VSI, viscerosomatic index*.

### Blood Chemical Properties

The hematological characteristics in *B. gonionotus* are presented in Table 4. The Hct, Hb, and RBC contents of blood were significantly higher (*p* < 0.01) in fish fed with the FM 40 and FM 20 + FSM 20 diets compared to the SM 40 diet. Fish fed with the FSM 40 and SM 40 diets showed significantly (*p* < 0.01) higher blood glucose levels compared to the FM 40 and FM 20 + FSM 20 diets.

**Table 4:** Blood chemistry in silver barb (*Barbonymus gonionotus*) fed with different experimental diets for 90 days^a^.

**Parameters**	**Diets**				
	**FM 40**	**FM 20 + FSM 20**	**FM 20 + SM 20**	**FSM 40**	**SM 40**
Hct	34.5 ± 2.7^a^	33.2 ± 3.5^a^	31.2 ± 3.0^ab^	28.3 ± 2.9^ab^	26.1 ± 2.6^b^
Hb	8.5 ± 1.1^a^	8.1 ± 1.0^a^	6.9 ± 1.1^ab^	6.2 ± 0.9^ab^	5.9 ± 0.5^b^
RBC	3.9 ± 0.5^a^	3.4 ± 0.6^a^	3.3 ± 0.5^ab^	3.1 ± 0.4^ab^	2.5 ± 0.3^b^
Glu	7.1 ± 1.0^a^	6.9 ± 0.8^a^	7.5 ± 0.7^ab^	8.9 ± 0.7^b^	9.5 ± 0.8^c^

a *Values are presented with the mean ± SD of triplicate groups (n = 3) of fish where the values in each row with different superscripts are significantly different (p < 0.05)*.

*Hct, hematocrit (%); Hb, hemoglobin (100 g ml^−1^); RBC, red blood cell (×10^9^ ml^−1^); Glu, glucose (mmol L^−1^)*.

### Antioxidant Enzyme Activity

**Figure 1:**
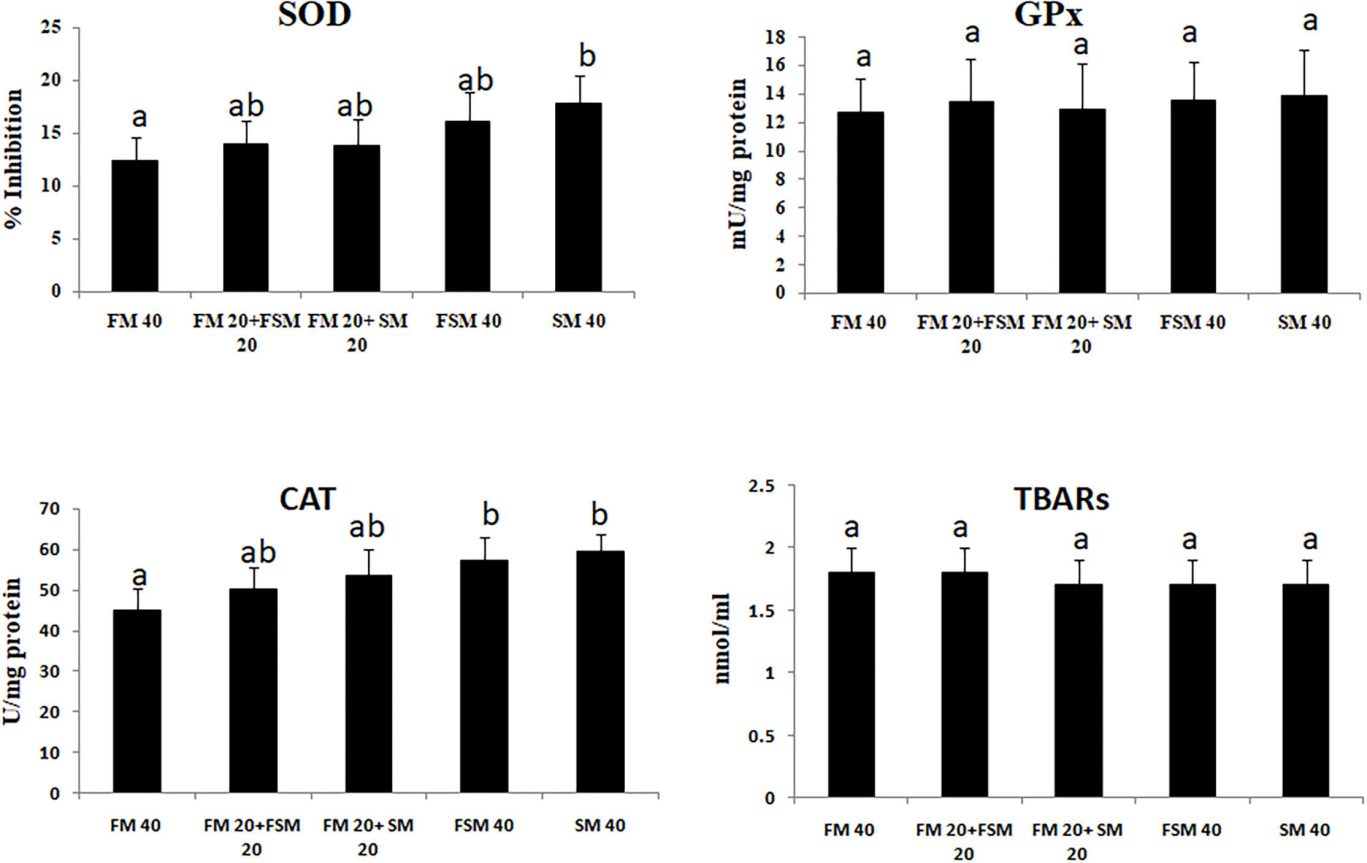
Antioxidant enzyme activities in *Barbonymus gonionotus* fed different levels of soybean meal for 90 days (*p* < 0.01). ^a, b^Values are mean±SD of triplicate groups (*n* = 3) of fish where the different letters are significantly different (*p* < 0.05).

Fish fed with the SM 40 diet showed significantly (*p* < 0.01) higher inhibition of SOD and CAT activities compared to the FM 40 diet. The GPx activity and TBARS have not differed significantly among the different diet groups (Figure 1).

### Histology of Anterior and Posterior Intestine

**Figure 2:**
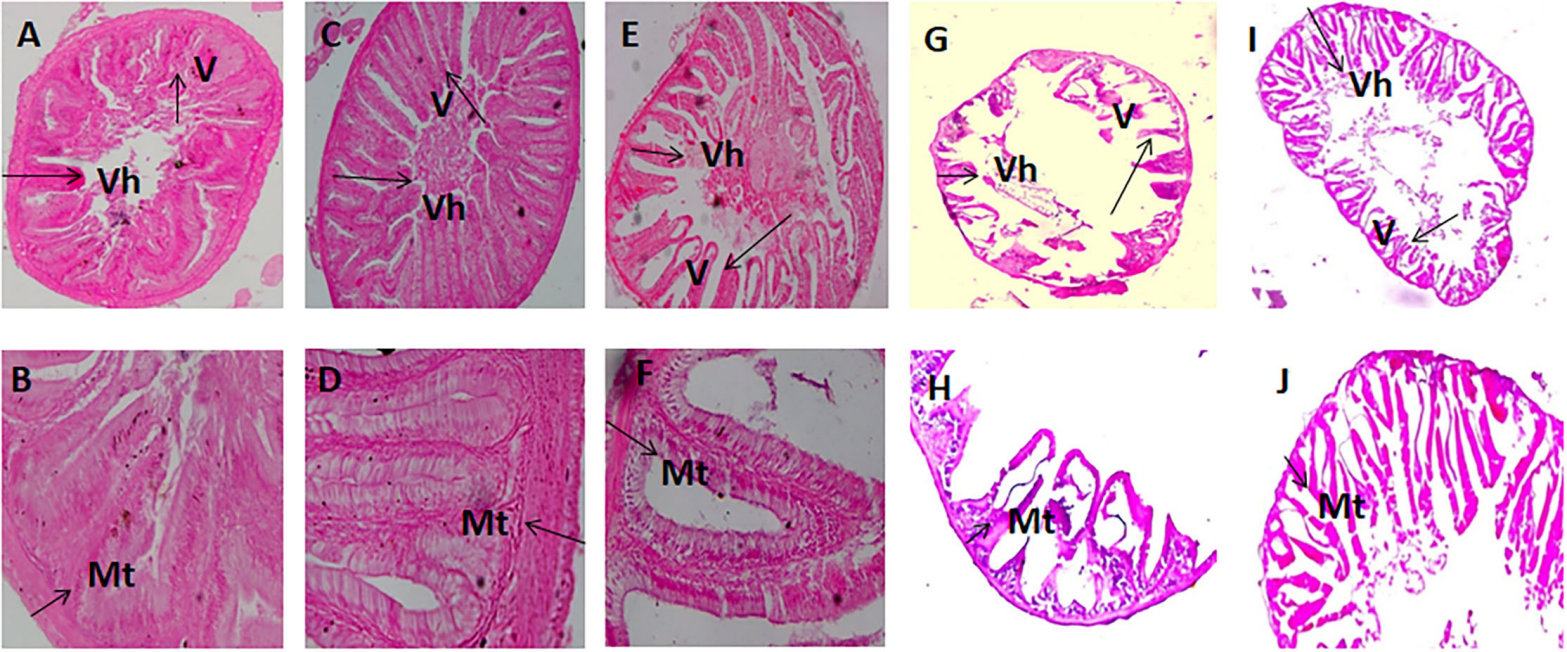
Intestinal segments obtained from fish fed with diet SM 40 **(A,B)**, FSM 40 **(C,D)**, FM 20 + SM 20 **(E,F)**, FM 20 + FSM 20 **(G,H)**, and FM 40 **(I,J)**. **(A,C,E,G,I)** (×10) and **(B,D,F,H,J)** (×40). V, villus; Vh, villus height; Mt, muscular thickness.

The histomorphic measurements of anterior and posterior intestinal Vh and muscular thickness are summarized in Table 5. Fish fed with the FM 40, FM 20 + FSM 20, and FM 20 + SM 20 diets showed significantly increased (*p* < 0.01) Vh and muscular thickness compared to the FSM 40 and SM 40 diets in anterior and posterior intestines of the fish (Table 5). However, in the posterior intestine, fish fed with the FSM 40 diet showed significantly higher Vh than the fish fed with the SM 40 diet. Fish fed with the FM 20 + SM 20, FSM 40, and SM 40 diets showed an intact mucosa, tidy villus folds, and enterocytes in regular shape; no inflammation, no lesions, and no loss of epidermal integrity were observed in the intestine of fish (Figures 2A–F). Fish fed with the FM 20 + FSM 20 and FM 40 diets showed tidy villus folds and little decrease in Vh of intestine compared with the SM 40 diet (Figures 2G–J).

**Table 5:** Histomorphological measurements of the anterior and posterior intestine of *Barbonymus gonionotus* fed with different experimental diets for 90 days^a^.

**Parameters**	**Diets**				
	**FM 40**	**FM 20 + FSM 20**	**FM 20 + SM 20**	**FSM 40**	**SM 40**
**Anterior intestine**					
Vh (μm)	558.7 ± 26.5^a^	550.7 ± 25.8^a^	494.3 ± 21.4^a^	310.0 ± 14.2^b^	290.3 ± 10.9^b^
Mt (μm)	149.3 ± 9.0^a^	154.7 ± 10.2^a^	190.7 ± 9.1^b^	225.7 ± 9.3^c^	344.7 ± 16.4^d^
**Posterior intestine**					
Vh (μm)	294.0 ± 20.0^a^	284.3 ± 21.5^a^	272.7 ± 17.0^a^	154.0 ± 13.5^b^	110.2 ± 12.9^c^
Mt (μm)	126.8 ± 6.8^a^	132.0 ± 7.8^ab^	154.3 ± 9.3^b^	202.3 ± 12.7^c^	281.7 ± 13.4^d^

a *Values are presented with the mean ± SD of triplicate groups (n = 3) of fish where the values in each row with different superscripts are significantly different (p < 0.05)*.

*Vh, villus height (μm); Mt, muscular thickness (μm)*.

### Digestive Enzyme Activities in Fish

Fish fed with the FM 40 and FM 20 + FSM 20 diets showed significantly (*p* < 0.05) higher protease, lipase, and amylase activities in the anterior intestine compared to the SM 40 diet (Table 6). In the posterior intestine, protease activity was significantly (*p* < 0.05) higher in fish fed with the FM 40 diet, whereas lipase and amylase activity were increased significantly (*p* < 0.05) in the FM 40 and FM 20 + FSM 20 diet groups (Table 6).

**Table 6:** Digestive enzymes activity (U mg^−1^ protein) in the anterior and posterior intestine of *Barbonymus gonionotus* fed with different experimental diets for 90 days^a^.

**Enzyme activity (U mg protein)**	**Diets**				
	**FM 40**	**FM 20 + FSM 20**	**FM 20 + SM 20**	**FSM 40**	**SM 40**
**Anterior intestine**					
Protease	47.3 ± 6.4^a^	46.1 ± 5.5^a^	42.7 ± 5.7^ab^	37.2 ± 4.4^ab^	30.7 ± 5.4^b^
Lipase	119.4 ± 12.1^a^	116.2 ± 11.8^a^	98.9 ± 10.3^ab^	95.5 ± 8.8^ab^	87.4 ± 7.2^b^
Amylase	2.7 ± 0.2^a^	2.5 ± 0.1^a^	1.9 ± 0.2^b^	1.8 ± 0.1^b^	1.6 ± 0.2^b^
**Posterior intestine**					
Protease	28.5 ± 3.1^a^	23.4 ± 3.2^ab^	21.6 ± 2.1^ab^	20.3 ± 4.8^ab^	18.8 ± 3.4^b^
Lipase	79.7 ± 6.8^a^	78.3 ± 5.3^a^	72.3 ± 6.3^ab^	66.5 ± 8.2^ab^	61.8 ± 6.1^b^
Amylase	1.6 ± 0.1^a^	1.4 ± 0.1^a^	0.9 ± 0.1^b^	0.8 ± 0.1^b^	0.8 ± 0.1^b^

a *Values are presented with the mean ± SD of triplicate groups (n = 3) of fish where the values in each row with different superscripts are significantly different (p < 0.05)*.

## Discussion

The current study revealed that FM in diets for *B. gonionotus* can be partially replaced by FSM and SM with a positive impact on growth performance, physiological, and hematological parameters. Besides the nutritional composition of the diet, temperature, dissolved oxygen, and pH also favor fish growth (26). In the present study, the culture condition for the growth of *B. gonionotus* was suitable with temperature 25–30°C, pH 6–7 and dissolved oxygen 8–9 ppm.

### Growth Performance of Silver Barb

In this study, *B. gonionotus* showed better tolerance to the FSM and SM diets, which resulted that the FSM and SM could replace at 50% of FM without compromising body WG and SGR (%). Similar results also have been reported in the case of silver barb (27), sea bass (28), tilapia (29), and even in the crustacean, juvenile Pacific white shrimp (30) without negative effects on the growth following the partial replacement of FM by SM or FSM. Fish fed with the FSM 40 and SM 40 diets were recorded with significantly (*p* < 0.05) lower weight gain and SGR. The results indicated that 100% replacement of FM with FSM (FSM 40) or SM (SM 40) in the diets of fish was failed to achieve significantly higher or the same growth performance of fish fed with the partially replaced FM with FSM or SM in the diets. Likewise, HSI and VSI were found to be decreased in fish fed with the FSM 40 and SM 40 diets. However, fish fed with the FM 20 + FSM 20 and FM 20 + SM 20 diets showed no significant differences in HSI and VSI compared to fish fed with the FM 40 diet. The results indicate that the liver and intestinal conditions of fish fed the 50% of FM replaced with FSM or SM diets were unaltered.

### Hematology of Silver Barb

The hematological parameters are the physiological reflector of general fish health and are reported to be affected by a range of factors including environmental conditions, species, size, age, and also dietary regime (31). In this study, blood parameters were considered to be within the normal range except in fish fed with the FSM 40 and SM 40 diets as compared to the previous findings for freshwater omnivore species like tilapia in the same condition (32). Results of the present study showed that the blood chemical parameters, such as Hct, Hb, and RBC, were significantly higher in the fish fed with the FM 20 + FSM 20 diets than the fish fed with the SM 40 diet, which attributed to the beneficial effects of the FM 20 + FSM 20 diet compared to the SM 40 diet, and a high inclusion rate of SM (SM 40) was stressful for the fish. Furthermore, 50% replacement of FM with FSM or SM and 100% replacement of FM with FSM in the diets of fish showed no significant changes in Hct, Hb, and RBC levels, which indicate the health status of fish was not affected by the FSM diet even at the higher inclusion rate. The results are consistent with other previous studies, which mentioned that dietary FSM had increased the nonspecific immune responses in addition to blood chemistry, such as Hct and Hb, in Nile tilapia (33). On the other hand, the plasma glucose level followed a decreasing trend against the dietary inclusion of SM, which indicates that the full replacement of FM with SM is hazardous and not recommended for silver barb fish. A similar decreasing trend of plasma glucose content has also been reported in the case of Amberjack Juveniles (34) fed with the high SM diet.

### Antioxidant Enzyme Activities in Silver Barb

Antioxidant enzyme protection of vertebrates against reactive oxygen species (ROS) damage is obvious (35), which indicates the health conditions of an animal in response to several external stimuli (36). In the present study, SOD and CAT were significantly increased in fish fed with the SM 40 diet. There were no significant differences in SOD levels in fish fed with the FM 40 and FSM 40 diets, which indicates the less stress effects of FSM 40 than the SM 40 diet. However, in the present study, CAT activities indicate that 50% of FM could be replaceable by FSM or FM based on stress conditions in fish. Similarly, it has been documented that the dietary SM supplementation resulted in increased antioxidant activities (SOD, CAT, GPx, GOT, and GPT) in milk fish (36). Oxidative stress in terms of elevated levels of GPx and TBARS is generally an indication of liver injury (37). However, in this study, GPx and TBARS are relatively insensitive to the SM and thus suggested that less oxidative stress has occurred with a balance between the ROS generation and oxidative stress that was not pronounced in fish fed with the 100% replacement of FM with FSM or SM in the diets. Therefore, the results of the present study demonstrated that the fish liver was not affected by 100% replacement of FM with FSM or SM diets based on GPx and TBARS activities.

### Histomorphology of Intestine in Silver Barb

The plant protein inclusion in the fish diet has effects on gut morphology, particularly on the intestinal length. It is well-established that the plant-originated diet has been digested in the intestine and increased the volume of the intestine due to the presence of a large amount of fiber. In the present study, the FSM 40 and SM 40 diets showed significantly higher (*p* < 0.05) intestinal length (Table 3). Dietary inclusion of SM has also been reported to increase the length of the intestine in Atlantic salmon (*Salmo salar*) (38) and Nile tilapia (*Oreochromis niloticus*) (14). On the contrary, animal protein in the diet has functional effects on the liver (39). In the present study, HSI was significantly increased in the FM-added diets. Furthermore, the replacement of FM with FSM or SM resulted in a significant reduction of Vh in both anterior and posterior intestine of silver barb as the villi increased the surface area for the absorption of plant-derived nutrients. Intestinal Vh of Japanese sea bass, giant grouper, and trout was significantly reduced after having dietary plant protein concentrate in their diets (40–42). On the contrary, muscular thickness (Mt) in the anterior and posterior intestine of the silver barb was significantly increased with concomitant replacement of FM by FSM or SM in the diets. These structural changes in both parts of the intestine were also consistent with a previous study (13, 43). In the present study, the integrity of the digestive tract was not affected by FSM- or SM-based protein-sourced diets.

### Digestive Enzymes Activities in Silver Barb

There are an array of factors including feeding habits, feed preferences, principal dietary nutrients, and ANFs that play an important role in the digestive physiology in fish and also reflect the enzyme profile and its capability to hydrolyze, absorb, and assimilation (4). In the present study, the replacement of the total amount of FM with SM resulted in significantly reduced protease, lipase, and amylase enzyme activities in the anterior and posterior intestines of fish. In agreement with our study, high-level incorporation of soy products in the diet was also found to show reduced endogenous digestive enzyme activities in hybrid tilapia (44), Chinese sucker (*Myxocyprinus asiaticus*) (4), and Japanese seabass (*Lateolabrax japonicus*) (45). The reduction of digestive enzyme activities could have been caused by the inferior feed utilization by fish and ANFs in SM, which inhibit enzyme activities in fish (46). Interestingly, in this study, we found that the dietary FSM inclusion up to 100% replacement of FM showed no significant differences in protease and lipase activities, which indicate the protein and lipid digestions were not hampered in fish fed with the fully replaced FM with FSM in the diet. Furthermore, no significant difference was found in amylase activities in fish fed with the FM 40 and FM 20 + FSM 20 diets, which indicate carbohydrate digestion was not affected in fish fed up to 50% replacement of FM with FSM in the diet.

## Conclusions

In conclusion, the findings of the present study revealed that up to 50% of FM could be replaced by FSM or SM in the diets without significantly affecting the growth, gut health, digestive physiology, antioxidant enzyme activities, and blood chemical parameters of the juvenile silver barb. Therefore, the findings of the present study can be a model experiment to understand the appraisals of SM in the diet of other omnivores including monogastric farm animals.

## Data Availability Statement

The original contributions presented in the study are included in the article/supplementary files, further inquiries can be directed to the corresponding author/s.

## Ethics Statement

The animal study was reviewed and approved by the Animal Welfare and Experimental Ethics Committee (AWEEC/BAU/2020,21) of Bangladesh Agricultural University, Mymensingh, Bangladesh.

## Author Contributions

HJ planned the experiment, determined the growth, somite indices, digestive enzyme activities, and drafted the final article. IT determined the hematological parameters, antioxidant enzyme activities, and final drafting of the manuscript. WQ analyzed the antioxidant enzyme activities and finalized the manuscript. MM did the major statistical analysis and helped in drafting. MP and RA determined histological analysis and helped to set the experiment. AO helped in the final drafting and checking the grammar of the manuscript. TM helped in review and editing and finalizing the manuscript. ZH critically supervised and helped in experimental planning with the addition of manuscript drafting. All authors read and approved the final manuscript.

## Funding

This research work was funded from the special grants by the Ministry of Science and Technology, Government of the People's Republic of Bangladesh, Dhaka, Bangladesh under a project (2018/MoST-67) in favor of ZH (ORCID No. 0000-0002-9144-9967).

## Conflict of Interest

The authors declare that the research was conducted in the absence of any commercial or financial relationships that could be construed as a potential conflict of interest.

## Publisher's Note

All claims expressed in this article are solely those of the authors and do not necessarily represent those of their affiliated organizations, or those of the publisher, the editors and the reviewers. Any product that may be evaluated in this article, or claim that may be made by its manufacturer, is not guaranteed or endorsed by the publisher.
